# Biphasic Packing of DNA and Internal Proteins in Bacteriophage T4 Heads Revealed by Bubblegram Imaging

**DOI:** 10.3390/v12111282

**Published:** 2020-11-10

**Authors:** Weimin Wu, Naiqian Cheng, Lindsay W. Black, Hendrik Dietz, Alasdair C. Steven

**Affiliations:** 1 Laboratory of Structural Biology Research, National Institute of Arthritis Musculoskeletal and Skin Diseases, National Institutes of Health, Bethesda, MD 20892-8025, USA; weimin.wu@nih.gov; 2 Department of Biochemistry and Molecular Biology, University of Maryland Medical School, Baltimore, MD 21201-1503, USA; lblack@som.umaryland.edu; 3 Physics Department, Technische Universität München, 85748 Garching-bei-München, Germany; dietz@tum.de

**Keywords:** cryo-electron microscopy, DNA packaging, coaxial spool, DNA origami, radiation damage, bubblegram

## Abstract

The virions of tailed bacteriophages and the evolutionarily related herpesviruses contain, in addition to highly condensed DNA, substantial quantities of internal proteins. These proteins (“ejection proteins”) have roles in scaffolding, maturational proteolysis, and cell-to-cell delivery. Whereas capsids are amenable to analysis at high resolution by cryo-electron microscopy, internal proteins have proved difficult to localize. In this study, we investigated the distribution of internal proteins in T4 by bubblegram imaging. Prior work has shown that at suitably high electron doses, radiation damage generates bubbles of hydrogen gas in nucleoprotein specimens. Using DNA origami as a test specimen, we show that DNA does not bubble under these conditions; it follows that bubbles represent markers for proteins. The interior of the prolate T4 head, ~1000 Å long by ~750 Å wide, has a bubble-free zone that is ~100–110 Å thick, underlying the capsid shell from which proteins are excluded by highly ordered DNA. Inside this zone, which is plausibly occupied by ~4 layers of coaxial spool, bubbles are generated at random locations in a disordered ensemble of internal proteins and the remainder of the genome.

## 1. Introduction

The packing of DNA in the capsids of tailed bacteriophages has long attracted interest in the context of mechanisms for the condensation of large DNA molecules [1,2,3]. In this state, the DNA is compressed to high density (~450 mg/mL), from which it must be released to initiate the next cycle of infection. A number of arrangements have been proposed. These include “coaxial spool” models, according to which the DNA is wound in nested shells [4,5], as well as “spiral fold” models in the case of T4 and lambda [6,7]. In the phage T7 system, cryo-electron microscopy (cryo-EM) has yielded strong evidence in support of a coaxial spooling model, with the spooling axis parallel to the portal axis [8]. Cryo-EM data also support spool models for, among others, phages HK97 [9] and P22 [10,11], both of which, like T7, have isometric T = 7 capsids, and for the phage-like herpes simplex virus (isometric, T = 16) [12,13,14].

T4 is a well-studied phage that differs from T7 in several respects. First, it has a much larger genome, ~170 kbp vs. ~40 kbp. Second, the wild-type T4 capsid differs in size and shape, which corresponds to an icosahedron extended along the portal axis, with triangulation numbers of T = 13 for the end-caps [15] and Q = 20 for the mid-section [16]. The well-characterized genetics of T4 make it possible to perturb the prolate head structure in potentially informative ways. Certain mutations in the major capsid protein gp23 result in isometric capsids [17], and mutations in the vertex protein gp24 yield massively elongated “giant” heads [18,19]. 

We have now extended the cryo-EM analysis of T4 with “bubblegram” imaging. In this approach, multiple successive exposures, “dose series”, are recorded [20,21]. This eventually results in the generation of hydrogen gas bubbles [22] that mark the locations of the molecules of origin. This information can guide interpretation of structural information from the first, minimally damaged, exposure. However, without further information, it is not possible to distinguish between protein density and DNA density. To resolve this ambiguity, we performed bubblegram imaging on a protein-free test specimen that is suitably dense, i.e., close to the density of DNA in a viral nucleocapsid. For this purpose, we used a 4.6 MDa DNA origami [23], and found that it did not bubble at electron doses well beyond the bubbling threshold for protein. We concluded that the bubbles observed in irradiated T4 and other phages derive from protein. These data provide further support for a biphasic model in which several, approximately four, coaxial coils of DNA surround a disordered nucleoprotein interior.

## 2. Materials and Methods

### 2.1. Preparation of Virions and Recording of Bubblegrams

Wild-type T4 phage and mutant strains were propagated, and the corresponding virions were isolated as previously described [3]. For cryo-EM, 3 μL drops of phage-containing suspension were applied to holey carbon films, then reduced to thin films by blotting, and vitrified by plunging into liquid-N_2_-cooled liquid ethane. Electron microscopy was performed essentially as described by Cheng et al. [24]. The micrographs were recorded on film. Dose series were recorded as multiple 1 s exposures, 10 s apart, usually at an illumination corresponding to 15 el/Å^2^ per exposure, sometimes slightly more. In a given dose series, these images are referred to below as exposure 1, exposure 2, etc. Bubblegram imaging of P22 virions, kindly made available by Dr. C. Teschke (Dept. of Molecular and Cell Biology, University of Connecticut, Storrs), has been described [25].

### 2.2. Estimation of DNA Content of a T4 Virion in Coaxial Spool Models

We approximated the outermost spool as a prolate ellipsoid of revolution with a major axis of 1000 Å and a minor axis of 750 Å. Its surface area was calculated using an Internet-based mathematical routine (EasyCalculation.com). Alternatively, the same area could be calculated for a single ribbon, 25 Å wide, to obtain the equivalent length of B-form DNA. Dividing that length by 3.4 gave the estimated number of base pairs in this shell. For the second and third shells, and so on, the calculation was repeated with the axes reduced by 50 Å at each step.

### 2.3. Image Averaging and Difference Images

In a given dose series, second and later exposures were aligned with the first exposure by cross-correlation, except for a few ambiguous cases that were handled by manually picking three reference points on each image at the same easily distinguished locations. In all, 15 dose series encompassing 564 particles were used for this analysis. After alignment, averaged images were calculated, as were difference images between the 1st exposure and Nth exposure.

## 3. Results and Discussion

### 3.1. DNA Complexes Are Bubbling-Reluctant: Origami Do Not Bubble after a Dose of 450 el/Å^2^

In order to distinguish proteins from DNA in a nucleoprotein particle through the use of bubblegram imaging, it is essential to know whether DNA bubbles when subjected to doses known to cause the bubbling of protein. If DNA does not bubble, it can be concluded that observed bubbles derive from proteins. We addressed this issue empirically by imaging, at progressively higher doses, a protein-free DNA particle that resembles an encapsidated genome in terms of size, shape, and degree of compaction. The most suitable particle that we found for this purpose was a DNA origami consisting of 15,238 nt with a molecular mass of 4.8 MDa [23]. This particle is stabilized by extensive hydrogen bonding into a compact globular form some 25–35 nm across, depending on viewing direction.

**Figure 1:**
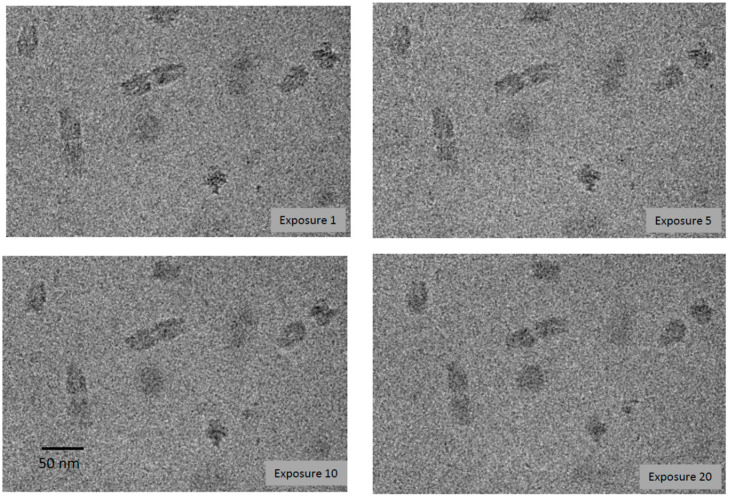
Cryo-electron micrographs from a dose series of images of DNA origami.

**Figure 2:**
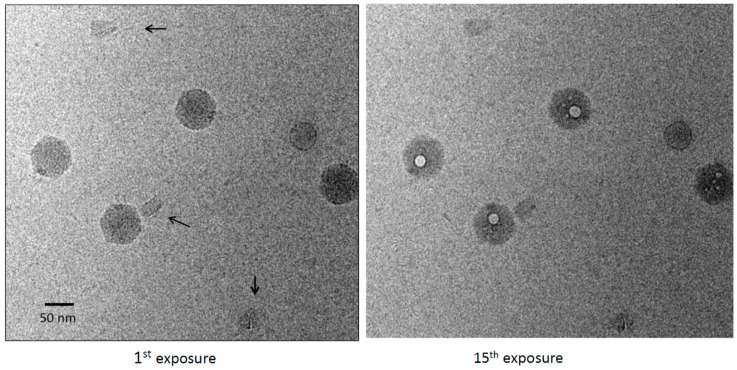
First and 15th exposures from a dose series of a mixture of DNA origami and bacteriophage P22. Origami are marked with black arrows in the left-hand panel. The virions show large primary bubbles (right-hand panel), plus a few small secondary bubbles, whereas the origami have no bubbles.

Vitrified specimens were imaged in dose series of up to 30 sequential exposures, corresponding to a cumulative electron dose of ~450 el/Å^2^. The origami particles showed no sign of bubbling. The only effect of this treatment was a modest degree of blurring (Figure 1). The cumulative dose applied was at least threefold higher than the bubbling thresholds we have observed in several other viruses [20,24,25,26]. Exposure 10, corresponding to a dose of ~150 el/Å^2^, already elicits copious bubbling of these specimens. Control experiments were performed in which the origami was mixed with bacteriophage P22. Bubbling in the virions was well-established by exposures 9 to 10 [25] (Figure 2). As expected, each virion developed a large primary bubble (occasionally, two bubbles) nucleated in the portal protein, in addition to a few smaller secondary bubbles. By exposure 15, the primary bubbles reached a diameter of ~160 Å. No bubbling of the origami was observed, even in exposure 30.

### 3.2. Bubblegrams of Bacteriophage T4 Heads

**Figure 3:**
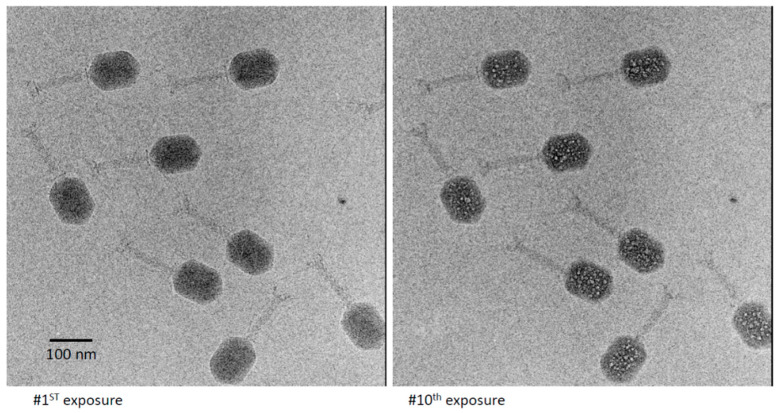
First and 10th exposures from a dose series of cryo-electron micrographs of a field of T4 virions. In the 10th exposure, multiple bubbles are seen in each virion, which are randomly distributed except for exclusion from a peripheral zone about 110 Å thick.

T4 virions were prepared and imaged in dose series similar to those described above. When confined to a thin film, the virions orient so as to present side-views. This appears to reflect maneuvering of the tailed virion, which is highly asymmetric, so as to remain hydrated. Two members of a representative dose series are shown in Figure 3. As expected, the early exposures resulted in only a moderate degree of blurring. At exposure 8, the first small bubbles appeared. These bubbles grew, and additional bubbles were nucleated in subsequent exposures. The bubblegram was well developed and maximally informative as to the locations of bubbling-competent material in exposures 10 and 11. At this point, there were 20 to 30 bubbles per virion of various sizes, but these were typically 30–50 Å across, and generally much smaller than the primary bubbles generated at similar doses in T7 [24] or P22 virions [25]. A movie illustrating the development of bubbles in T4 virions is shown in Supplementary Material Movie S1. Eventually, the bubbling became so extensive that the yield of site-specific information was reduced through, for example, bubble-merging. 

**Figure 4:**
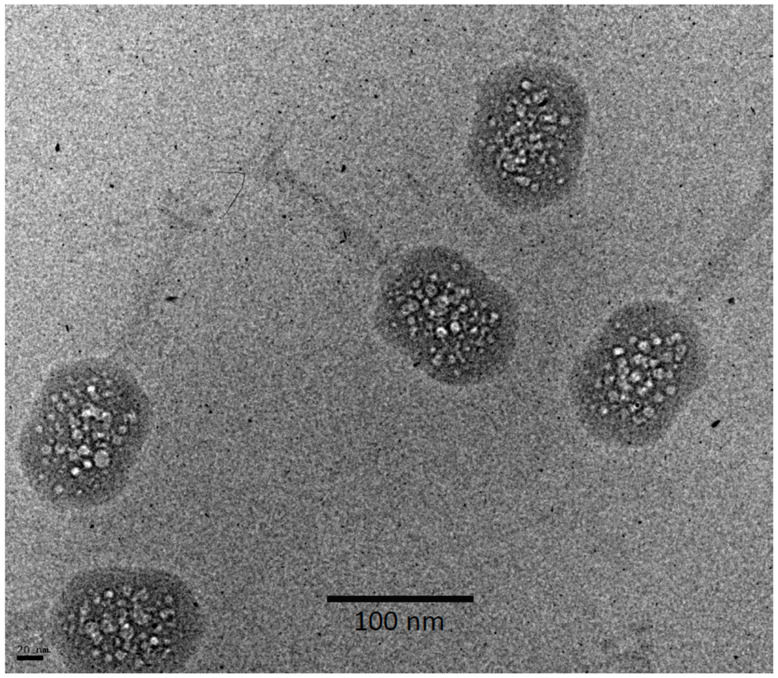
Cryo-electron micrograph of a field of T4 virions exhibiting more advanced bubbling. This was the 8th exposure of an alt-minus mutant imaged at 16.5 el/Å^2^ per exposure. Wild-type virions imaged under similar conditions showed no significant difference.

As recalled above, in the bubbling patterns of phages T7 or P22, a large primary bubble is generated in the protein core on the interior side of the portal vertex, together with a few small secondary bubbles. A striking feature of T4 bubbling is the exclusion of bubbles from a zone about 100–110 Å thick, underlying the capsid shell (Figure 3). This exclusion zone is maintained, even at higher doses (Figure 4). Apart from this zone, the distribution of bubbles appears random throughout the interior of the T4 head.

**Figure 5:**
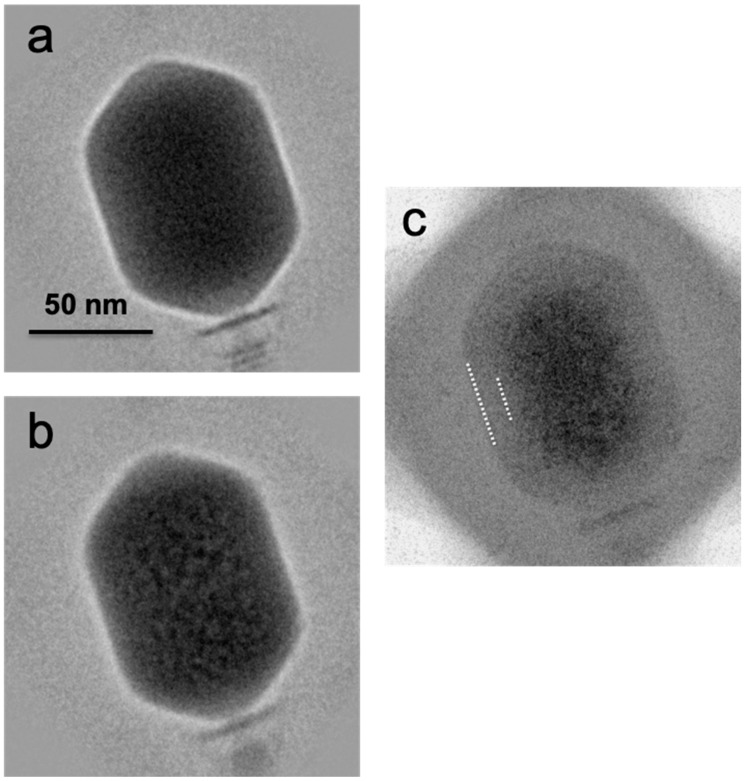
Averaged images of first exposures (**a**) and 10th exposures (**b**) calculated from cryo-electron micrographs of bacteriophage T4 heads. In **(c**) is the averaged difference image obtained between the 1st exposure (low dose) and the 10th exposure (bubbling dose). Positive image densities are shown as darker. White dashes mark the approximate limits of the bubble-free zone.

To further demarcate the exclusion zone, we calculated averaged images and difference images. Examples are shown in Figure 5. To obtain such an image, the local steps in density from pixel to pixel were calculated, averaged, and mapped. Around the edge of a bubble, these steps were much larger than the case for a non-bubbling particle. Essentially, this imaging procedure acted as a high-pass filter. The density map obtained by combining the data from many particles gave an account of the overall distribution of bubbles (Figure 5c). A similar conclusion was reached from the “variation images” in Supplementary Material Figure S1. These data showed that the exclusion zone is essentially uniform in thickness (~100–110 Å). Inside the exclusion zone, bubbles are randomly distributed and there are no “hot spots” like those that generate the primary bubbles of T7 [24], P22 [25], or φKZ [20]. 

### 3.3. Bubblegram Imaging Supports a Biphasic Model for Wild-Type (Prolate) T4 Heads

The thickness of the outer region (bubble-free zone) is readily explained as consisting of approximately four coils of spooled DNA. This accounts for about 54% (see Materials and Methods) of the 170 kbp genome. Interior to that is the second region, which accommodates the remaining 46% or so of the genome (mass, approximately 35 MDa) and most if not all of the internal proteins—gp22*, gpalt, gp67, IPI, IPII, and IPIII. This adds up to about 18 MDa, contributed by ~1370 subunits [2]. The IPs enter the head at the stage of prohead assembly, due to their possessing a ~10 amino acid N-terminal capsid targeting sequence (CTS). The CTS is able to encapsidate virtually any protein to which it is attached [27]. Once packaged, the IP subunits are detached from the CTS by a head maturation protease [28]. 

As yet, we are not able to differentiate the respective contributions to bubbling that the various internal proteins make. However, we note that the thin capsid shell did not bubble under the experimental conditions used, and we attribute this behavior to hydrogen generated in the capsid diffusing too rapidly from its site of origin for bubbles to form [24].

The bubblegram data presented here strongly support the coaxial spool scenario, in that the thickness and location of the exclusion zone match what is expected for some four layers of a coaxial spool at 25 Å per layer. It is the orderly close-packing of DNA duplexes in the spool that excludes the internal proteins from it, accounting for the absence of bubbles. Alternatively, the exclusion zone may represent a bubble escape zone that allows hydrogen bubbles to escape through the nearby porous capsid wall. Inside the spool is a less ordered region occupied by randomly distributed proteins and DNA. The exclusion zone is uniformly thick all around the interior surface of the capsid. In this respect, T4 differs from T7 [24] and P22 [25], which both have exceptionally strong bubbling sites where their primary bubbles are generated, located on the interior side of their respective portals [29]. T4 has no such bubbling-prone site for internal proteins. 

Alternatively, support for a spiral fold structure in at least a part of encapsidated T4 DNA comes from data on CTS-tagged IPs that encapsidate staphylococcal nuclease, which preferentially cuts folded or bent DNA [30]. The encapsidated proteins were proposed to be embedded within the DNA [31] and to display high mobility, thereby allowing complete cutting of the encapsidated DNA to small fragments [28]. Prepackaged internal proteins also enhance the rate of DNA packaging [32]. This model may relate to the interior phase of the head, but the envisaged embedding of protein is hard to reconcile with the observed close packing of DNA in the exclusion zone.

In conclusion, we note that the coaxial spool scenario is also consistent with recent cryo-EM studies of herpesvirus nucleocapsids that directly visualized an outer region of three coaxially spooled shells [13] or (at least) a single such shell [14]. Of note, both studies ascertained that, although the HSV capsid is isometric, the spools are tilted at an angle of 15° to 20°, and they are left-handed.

## Supplementary Materials

The following are available online at https://www.mdpi.com/1999-4915/12/11/1282/s1, Movie M1: Time-lapse (dose lapse) animation combining micrographs from the following exposures: #1, 7, 8, 9, 10, and 11, Figure S1: “Variation images” and average transverse density profiles for 1st exposure (low dose) and 9th exposure (typical bubbling dose) of T4 heads calculated.
